# Subanaesthetic dose of esketamine during induction delays anaesthesia recovery a randomized, double-blind clinical trial

**DOI:** 10.1186/s12871-022-01662-0

**Published:** 2022-05-09

**Authors:** Cao Zhang, Jiangqin He, Qinye Shi, Fangping Bao, Jianhong Xu

**Affiliations:** Anaesthesia Department, The Fourth Affiliated Hospital Zhejiang University School of Medicine, No. N1, Shangcheng Road, Yiwu City, Zhejiang Province 322000 People’s Republic of China

**Keywords:** Esketamine, General anaesthetic recovery, Postoperative pain, Postoperative agitation, Postoperative nausea and vomiting

## Abstract

**Background:**

Esketamine is an antagonist of the N-methyl-D-aspartate receptor (NMDA receptor) that is widely used for multimodal analgesia. In addition to analgesia, sedation is another important effect of esketamine. However, data are limited regarding the sedation effect of esketamine during general anaesthesia. The objective of this study was to determine whether sedation with a subanaesthetic does of esketamine affects anaesthesia recovery.

**Methods:**

Fifty patients, ASA I to II patient scheduled to laparoscopic cholecystectomy, were randomly assigned to receive a single bolus of esketamine 0.2 mg kg-1 (esketamine group) or placebo (control group). Propofol, sufentanil and rocuronium were used during total intravenous anaesthesia. The patients’ time of recovery from anaesthesia, postoperative pain, postoperative nausea and vomiting, and postoperative agitation were analysed.

**Results:**

Data from 47 patients were analysed. The average time of anaesthetic recovery was 22.04 ± 1.48 min in the esketamine group(*n* = 23) and 17.54 ± 1.46 min in the control group(*n* = 24). The recovery time was significantly longer in the esketamine group. Postoperative pain in the PACU was lower in the esketamine group (NRS score range 0–2) than in the control group (NRS score range 0–3). There were no differences in postoperative nausea and vomiting, and postoperative agitation.

**Conclusion:**

Subanaesthetic doses of esketamine can reduce postoperative pain in the PACU but delay the aesthetic recovery during the laparoscopic cholecystectomy, without affecting postoperative nausea and vomiting, and postoperative agitation.

**Trial registration:**

The study was registered at the Chinese Clinical Trial Registry http://www.chictr.org.cn/ (Registration date: 20/11/2020; TrialID: ChiCTR2000040077).

## Background

Ketamine has been used since the *1960*s [1], and it has recently gained widespread used in pain management, neurology, and psychiatry [2–4]. A subanaesthetic dose of ketamine used as an adjuvant in general anaesthesia can improve postoperative pain and reduce the need for opioids [5, 6]. Ketamine is also widely used in painless gastroscopy as an analgesic [7, 8].

The S-enantiomer esketamine has been used in several European countries for decades and has been adopted in Chinese hospitals. Its anaesthetic effect is twice that of racemic ketamine [7]; therefore, lower clinical doses of esketamine are required, and side effects (such as nightmare, delirium, and agitation) are reduced [9].

Acute postoperative pain can be improved by using ketamine in the perioperative period [10–12] and taking advantage of the analgesic effect of subanaesthetic doses. However, the sedation effect of esketamine was ignored in these studies. To determine whether the use of subanaesthetic doses of esketamine during surgery would delay anaesthetic recovery, we conducted this research.

## Methods

### Ethics approval and consent to participate

Ethical approval was obtained from the Human Research Ethics Committee of the Fourth Affiliated Hospital of Zhejiang University School of Medicine at 08.07.2020(approval NO.: K2020086, Head: Prof. Dr. Zhu). The study was registered in the Chinese Clinical Trial Registry (ChiCTR2000040077, Principal investigator: Cao Zhang, Date of registration: 20/11/2020). Written informed consent was obtained from all subjects participating in the trial, and all methods were performed in accordance with the relevant guidelines and regulations. This manuscript adheres to the applicable CONSORT guidelines.

### Study design

This was a prospective, randomized controlled trial performed at anaesthesia department of the Fourth Affiliated Hospital of Zhejiang University School of Medicine from December *2020* to March 2021.

Patients scheduled for elective laparoscopic cholecystectomy were eligible for participation in this trial if their age was 18–65 years, they had an American Society of Anaesthesiologists Physical Status (ASA) I-II, and they were able to provide informed consent. The exclusion criteria were liver or kidney dysfunction, operation time greater than 2 h, intraoperative changes, body mass index (BMI) > 35 or < 18 kg*m^− 2^, history of neurological disease, long-term alcoholism, and intraoperative bleeding greater than 200 ml.

Included patients were allocated at a 1:1 ratio to the esketamine group (K group) or the placebo group (N group) and were randomized by nurse using a random number table created by statisticians using SPSS statistical software. This nurse gave the esketamine or placebo to the anaesthesiologists; thus, the anaesthesiologists were blinded. Patient data were recorded by the anaesthesiologists, and all information was collected by an independent researcher.

### Anaesthesia induction and maintenance

All patients received total intravenous anaesthesia (TIVA). Before the induction of anaesthesia, an intravenous cannula was inserted, and pulse oximetry (SpO_2_), non-invasive arterial blood pressure (NIBP) and ECG monitoring was performed. An infusion of Ringer’s solution was initiated. General anaesthesia was induced with midazolam (0.04 mg/kg), atropine (0.01 mg/kg), propofol (1.5 mg/kg), rocuronium (0.6 mg/kg) and sufentanil (0.5 μg/kg). Esketamine (0.2 mg*kg-1) or normal saline provided by the nurse was injected during induction before propofol administration. Anaesthesia was maintained using propofol and remifentanil. Propofol was continuously infused at a rate of 6 mg/kg*h, and the remifentanil infusion rate was 0.1–0.3 μg/kg*min. The anaesthesiologist adjusted the infusion rate of propofol and remifentanil according to the patient’s vital signs during the operation and recorded the vital signs every 5 min. At the end of the operation, all drugs were stopped, and tropisetron 5 mg was used.

In the postanaesthesia care unit (PACU), patients received standard monitoring (ECG, SpO_2_, NIBP and respiratory rate), and the nurses assessed the state of consciousness every 5 min by calling the patient’s name or tapping his or her shoulder. The time the patient’s eyes opened was recorded; the patients were also asked to shake their head, and if the patients breathed spontaneously on a regular basis, the respiratory rate (RR) was greater than 10 times/min, and the tidal volume was greater than 5 ml/kg, the patient’s tracheal tube was removed, and the time of removal was recorded as the recovery time. Pain assessment was performed at 15 min and 30 min after extubation; if numeric rating scale (NRS) scores were greater than 4, sufentanil 0.05 μg/kg was used.

### Experimental intervention

The patients in this study were divided into two groups: The esketamine group (K group) and the control group (N group). All patients were injected with 0.1 ml/kg experimental drugs during anaesthetic induction. The experimental drugs were prepared by independent nurses and comprised esketamine 2 mg/ml and normal saline. The nurses divided the patients by using a random number table created by a statistician using SPSS statistical software and gave the experimental drugs to the anaesthesiologists.

### Outcome assessment

The primary outcome was the anaesthetic recovery time. We defined the anaesthetic recovery time as the time from when the anaesthetic drugs were stopped to when the tracheal tube was removed. As described above, the patient needed to fully recover consciousness and respiratory function well before the tracheal tube could be removed. Indications for extubation were recovery of consciousness, ability to perform requested actions, steady breathing and RR greater than 10 times/min, and tidal volume greater than 6 ml/kg. The anaesthetic recovery time was recorded by PACU nurses.

Postoperative pain was another recorded outcome. The PACU nurses assessed the pain scales by NRS at the time when the patient recovered and 15 min and 30 min after the patient recovered. Postoperative agitation and postoperative nausea and vomiting (PONV) were also recorded.

### Statistical methods

#### Sample size calculation

The sample size was determined for the primary endpoint. According to the results of our previous study, the recovery time was 16.5 min in the control group. We anticipated a difference of 3.5 min in recovery time. With a power of 0.90 and a significance level of 0.05, the required sample size was calculated as 20 in each arm. Considering possible exclusions, we decided to include 25 patients in each group.

Statistical analyses were performed using the Statistical Package for Social Sciences (SPSS) software version 23.0 (SPSS, Inc., Chicago, Illinois, USA). All data were checked for normal distribution using the Kolmogorov-Smirnov test and histograms. For normally distributed, continuous variables, an independent Student’s t-test was used, and the variables are presented as the mean ± SD. A *P* value less than 0.05 was considered statistically significant. Not normally distributed data were compared using the Mann-Whitney U test, and data are presented as the median and interquartile range (IQR).

## Results

**Fig. 1:**
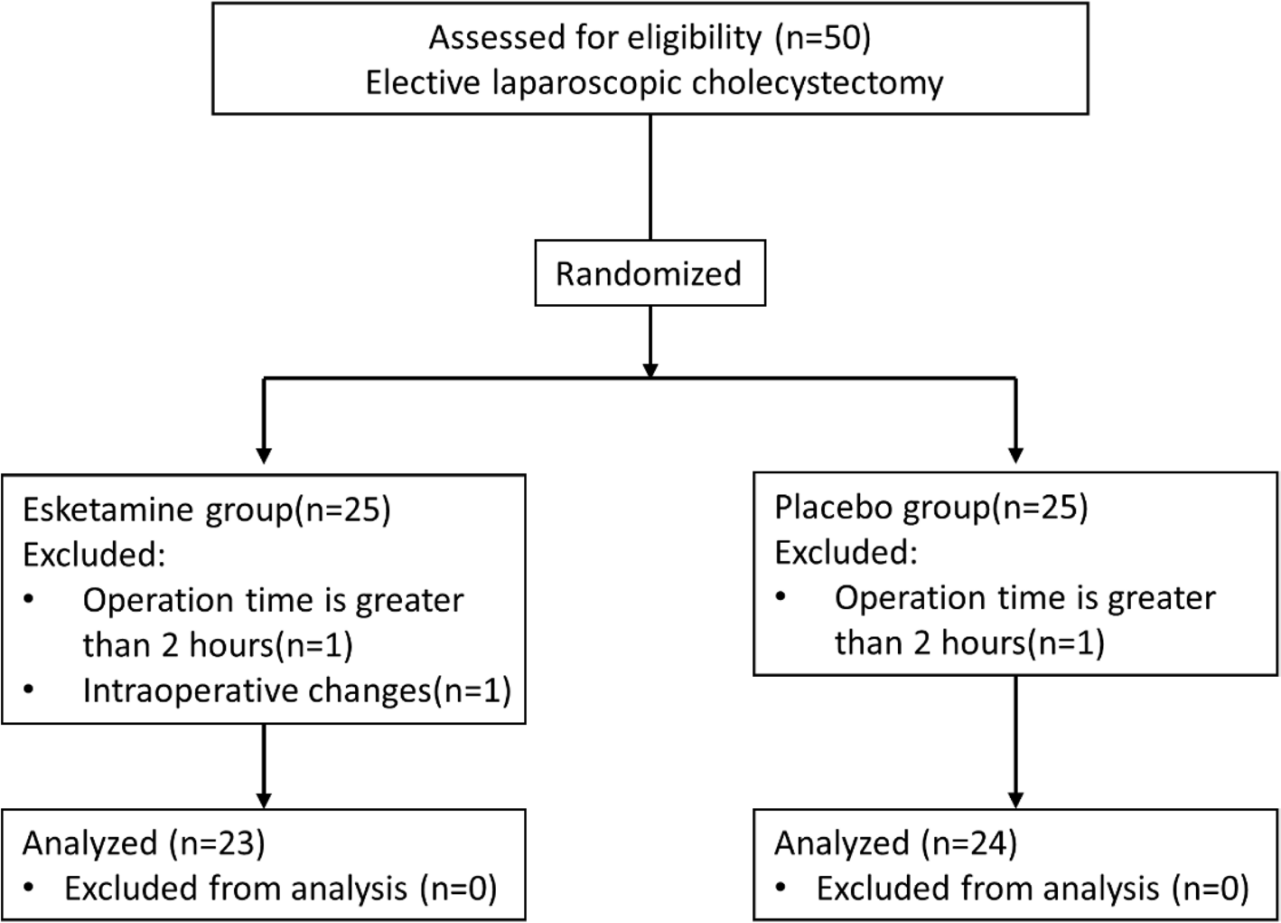
CONSORT flow diagram for the study. Esketamine group was administered esketamine (0.2 mg*kg^− 1^); Placebo group was administered normal saline as a control

From December 2020 to March 2021, a total of 50 patients were included in this study (Fig. 1) at the Fourth Affiliated Hospital Zhejiang University School of Medicine. Two patients with operation times longer than 2 h were excluded. One patient required a change of procedure and was excluded. Therefore, 47 patients (group K = 23; group *N* = 24) were analysed. Both groups were similar with respect to patient characteristics and operative duration (Table 1).

**Table 1:** Patients’ characteristics and baseline values

	Esketamine group(23)	Control group(24)	***P***-Value
Age (years)	51.57 ± 11.96	51.04 ± 8.35	0.863
Sex (male)	13 (56.5%)	11 (45.8%)	0.659
Weight (kg)	67.07 ± 8.45	66.52 ± 10.73	0.847
Hight (cm)	166.09 ± 7.06	163.08 ± 7.16	0.155
ASA PS	2 [1 to 2]	2 [1 to 2]	0.653
Hypertension	4 (17.4%)	3 (12.5%)	0.701
Diabetes	1 (4.3%)	2 (8.3%)	1.000
Duration procedure (min)	59.04 ± 21.12	58.75 ± 23.41	0.964
Baseline measurements			
SBP (mmHg)	138.15 ± 23.46	134.33 ± 14.89	0.514
DBP (mmHg)	80.77 ± 11.35	81.80 ± 9.62	0.739
SpO_2_ (%)	99 [97 to 100]	99 [96 to 100]	0.797
Heart rate (bpm)	81.92 ± 14.55	76.47 ± 9.13	0.134
Pain (NRS)	0 [0 to 1]	0 [0 to 0]	0.153
Nausea (Likert)	0 [0 to 0]	0 [0 to 0]	1.000
Vomiting (number)	0 [0 to 0]	0 [0 to 0]	1.000

### Primary outcome

**Fig. 2:**
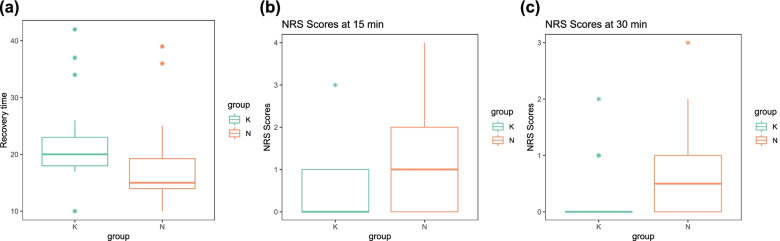
Anetshetic revovery time between ketamine group and control group and the postoperative pain in the PACU at the 15 min and 30 min after the tube removed. (**a**) there was significant difference in recovery time between groups. (**b**) the NRS scores were significant lower in ketamine group than control group. (**c**) the NRS scores were no significant different between groups at 30 min after revovery

We recorded the time at which the anaesthetic drugs were stopped and the tracheal tube was removed, and the difference between these two times was defined as the recovery time. The recovery time of the patients in group K (22.04 [95% CI: 18.97–25.12]) was significantly longer than that of the patients in the control group (17.54 [95% CI: 14.53–20.56]), *P* = 0.036 (Fig. 2).

### Secondary outcomes

Postoperative pain at 15 min after recovery was 1 (0 to 4) in the control group and 0 (0 to 1) in the esketamine group. At 30 min, the NRS score was 0 (0 to 1) in the control group and 0 (0 to 0) in the esketamine group. The NRS scores at 30 min of recovery were similar between the groups (*P* = 0.117), but at 15 min, the NRS scores were significantly lower in the esketamine group (*P* = 0.012) (Fig. 2). Two patients suffered moderate pain 15 min after recovery; they were given sufentanil 0.05 μg/kg, and their NRS scores dropped to 1 at 30 min. Only 1 patient (in the control group) experienced postoperative agitation, and no patient experienced postoperative nausea and vomiting (Table 2).

**Table 2:** Secondary outcomes

	Esketamine group(23)	Control group(24)	***P***-Value
Postoperative pain at 15 min			0.012
NRS: 0	14/23	8/24	
NRS: 1–3	9/23	14/24	
NRS: > 4	0/23	2/24	
Postoperative pain at 30 min			0.117
NRS: 0	18/23	12/24	
NRS: 1–3	5/23	12/24	
NRS: > 4	0/23	0/24	
postoperative agitation	0/23	1/24	1.000
postoperative nausea and vomiting	0/23	0/24	1.000

Values are expressed as number (positive)/number (total in the group). Esketamine group was administered esketamine (0.2 mg*kg^−1^); Placebo group was administered normal saline as a control

*NRS* Numeric rating scale

## Discussion

This is the first study that fouces on the effect of subanaesthetic doses of esketamine on recovery from anesthesia in a randomized controlled trial. The results show that using subanaesthetic doses of esketamine during anaesthesia induction prolongs the recovery time of patients under general anaesthesia undergoing elective laparoscopic cholecystectomy. Although the difference was statistically significant, the mean time was only 4.5 min longer when compared with the control group. In addition, no patients in either group experienced a delayed recovery (longer than 2 h). The longest recovery time in the esketamine group was 37 min.

The use of ketamine for analgesia is well established, the doses range from 0.15 mg/kg to 0.5 mg/kg [13, 14], esketamine also was used during anesthesia or PCIA for analgesia [11, 15]. Despite previous studies found that ketamine or esketamine could improve postoperative pain, which was consistent with our data. However research also found that intraoperative ketamine does not affect postoperative pain [2], Avidan MS found that ketamine (0.5 mg/kg or 1.0 mg/kg) after induction of anesthesia could not decrease the pain score during the postoperative 3 days by an international multicenter clinical research. We consider that intraoperative ketamine or esketamine only enhance analgesia over a short period, no longer than 24 h, as we attempted to address the postoperative pain just in the PACU.

Although ketamine or esketamine are widely used perioperative period, but little attention has been paid to their sedative effects. An animal research showed that subanesthetic ketamine use could accelerates recovery with isoflurane anesthesia by promoting arousal systems [16]. Our primary outcome was contrary to this literature. First this may be related to the drugs, propofol and midazolam were used during general anesthesia, not just isoflurane. Secondly, the opposite may due to different species. Furthermore, prolonged recovery time may be due to the sedative effect of esketamine.

Neurological symptoms are common and major side effects of esketamine, such as dreaminess, nightmares, delirium, etc. [17]. Lower doses of esketamine reduce the side effects [12], and intraoperative ketamine (0.5 mg/kg or 1 mg/kg) would not affect postoperative delirium [2]. In our research, no patient suffered postoperative agitation, PONV or psychiatric symptoms using 0.2 mg/kg esktamine during induction. Furthermore 0.2 mg/kg esktamine for elderly patients anaesthesia induction could maintain the stability of haemodynamics [18], which may have benefits for postoperative outcomes.

This study has limitations. First the sample size was small, and only Chinese adults were enrolled, so this may bias the findings; thus a larger multicentre and multiethnic research is warranted. Second elective laparoscopic cholecystectomy was a less stressful procedure, so the incidence of postoperative pain was low. Thus, the benefit of esketamine in analgesia should be considered in more different procedures.

## Conclusion

We found that the administration of a single subanaesthetic dose of esketamine could delay anaesthetic recovery in elective laparoscopic cholecystectomy, and could reduce postoperative pain.

## Data Availability

The datasets generated and analysed during the current study are available in the figshare repository, [https://figshare.com/s/f5e1bb2e49f99237a875]. All data are included in the manuscript.

## Abbreviations

NMDA: Receptor
N-methyl-D: aspartate receptor
ASA: American Society of Anaesthesiologists Physical Status
BMI: Body mass index
TIVA: Total intravenous anaesthesia
SpO_2_: pulse oximetry
NIBP: Non-invasive arterial blood pressure
RR: Respiratory rate
NRS: Numeric rating scale
PACU: Postanaesthesia care unit
PONV: Postoperative nausea and vomiting
